# Classification and prediction of drought and salinity stress tolerance in barley using GenPhenML

**DOI:** 10.1038/s41598-024-68392-w

**Published:** 2024-07-29

**Authors:** Mahjoubeh Akbari, Hossein Sabouri, Sayed Javad Sajadi, Saeed Yarahmadi, Leila Ahangar

**Affiliations:** 1 Department of Plant Production, Collage of Agriculture Science and Natural Resource, Gonbad Kavous University, Gonbad-E Kavus, 4971799151 Iran; 2 Horticulture-Crops Research Department, Golestan Agricultural and Natural Resources Research and Education Center, Agricultural Research, Education and Extension Organization (AREEO), Gorgan, 4969186951 Iran

**Keywords:** Drought, Salinity, Machine learning, Prediction, Classification, Barley, Plant biotechnology, Machine learning

## Abstract

Genetic and agronomic advances consistently lead to an annual increase in global barley yield. Since abiotic stresses (physical environmental factors that negatively affect plant growth) reduce barley yield, it is necessary to predict barley resistance. Artificial intelligence and machine learning (ML) models are new and powerful tools for predicting product resilience. Considering the research gap in the use of molecular markers in predicting abiotic stresses, this paper introduces a new approach called GenPhenML that combines molecular markers and phenotypic traits to predict the resistance of barley genotypes to drought and salinity stresses by ML models. GenPhenML uses feature selection algorithms to determine the most important molecular markers. It then identifies the best model that predicts atmospheric resistance with lower MAE, RMSE, and higher R^2^. The results showed that GenPhenML with a neural network model predicted the salinity stress resistance score with MAE, RMSE and R^2^ values of 0.1206, 0.0308 and 0.9995, respectively. Also, the NN model predicted drought stress scores with MAE, RMSE and R^2^ values of 0.0727, 0.0105 and 0.9999, respectively. The GenPhenML approach was also used to classify barley genotypes as resistant and stress-sensitive. The results showed that the accuracy, accuracy and F1 score of the proposed approach for salinity and drought stress classification were higher than 97%.

## Introduction

Barley (*Hordeum vulgare L.*) from the Poaceae family is one of the most important cereals for grain production, animal feed, and fermentation industries^1^. Barley is harvested as a profitable crop from over a hundred countries worldwide. Among the cereals, barley has the highest production rate after wheat, rice, and corn, accounting for nearly 145 million tons of world production in 2021^2^. The protein content of barley seeds is higher than that of rice, corn, and sorghum and is comparable to the proteins found in wheat grown under similar conditions^3^. The barley plant has a wide range of adaptations. This plant is cultivated where other grains do not grow well due to low rainfall, soil salinity, high altitude above sea level, and cold and hot weather. Abiotic stresses reduce the yield of agricultural products in the world. Meanwhile, salinity is one of the most important of these stresses^4^. In salinity stress, the metabolic disorders created cause damage to the plant and decrease the yield. These changes are different in different plant species and cultivars. Plants with appropriate tolerance to salt stress conditions achieve optimal performance. On the other hand, it is possible to identify, improve, and select salinity-tolerant species according to the genetic diversity between cultivars^5^. Another critical environmental stress that plants are exposed to is drought stress, and the importance of this stress is due to its pervasiveness. The incidence of drought, whether permanent or temporary, limits the natural growth and distribution of plants and ultimately reduces the yield of agricultural plants. Drought stress refers to a situation in which the water potential of the plant is reduced to such an extent that it disrupts the natural activities of the plant. The amount of water potential loss that leads to adverse effects depends on the type of plant, the growth stage, and the desired process. If the lack of water is severe, it can cause the complete stop of growth, the reduction or stop of photosynthesis, the disturbance of metabolic processes, and eventually, the death of the plant. Drought stress tolerance is a complex phenomenon involving several physiological and biochemical processes at cellular and whole levels. The organism is involved in different stages of plant development. Examples include reducing water loss by increasing stomatal resistance and water absorption by developing the root system and accumulating osmolytes. Barley is one of the most essential grains in developing countries, mainly where severe drought affects plant production. Barley has more efficient mechanisms against water shortage than other grains. However, the performance of this plant is limited by the dryness at the end of the season and the high temperature in the seed-filling stage^6^.

Early prediction of plant stress before it is visible to humans has essential implications for timely and cost-effective stress control and significantly impacts precision agriculture. Machine Learning (ML) models can detect and predict plant stresses^7–9^. ML models are widely used in agriculture and biotechnology for various purposes^10–13^. ML algorithms can learn from data. It aids in the interpretation of data, frequently after visualization. ML employs multiple strategies to address data difficulties, with the algorithm chosen based on complexity, variables, and ideal models. For plant stress prediction, ML models group genotypes into labeled classes. In this approach, models determine, for example, whether a genotype is tolerant or sensitive to a particular stress. This information can be used in breeding decision-making programs to select stress-resistant genotypes. Due to a research gap in using molecular marker features in predicting abiotic stresses, in this paper, we introduce a new approach called GenPhenML, which extracts relevant genotype and phenotype features to predict barley genotypes' tolerance to drought and salinity stresses. Genotype features were derived from quantitative trait loci (QTLs) and molecular markers analysis of barley. The GenPhenML evaluated several ML models such as Decision Trees (DTs), Linear Discriminant Analysis (LDA), Naive Bayes (NB), Support Vector Machine (SVM), K-Nearest Neighborhood (KNN), Random Forest (RF), Neural Networks (NN) and Gaussian Process Regression (GPR). The input of the models are phenotype and genotype features, and the output of the models is stress tolerance score (prediction) and resistance or sensitivity to stress (classification).

## Results

To emphasize the importance of molecular marker features, we investigated the performance of the GenPhenML with three scenarios. Only phenotype features were used as ML model input in the first one. In the second scenario, only genotype features were used, and in the third one, phenotype and genotype features were used to train ML models. The results are presented in two sections. In the first section, the results of the prediction of stress tolerance score, and in the second section, the results of the classification of stress tolerance are indicated.

### Prediction of drought and salinity stress score

Randomly partitioning phenotype and genotype data into the train and test dataset, the GenPhenML selects the best performing ML model after training the RF, SVM, NN, GP and DT model and test them with separate data from the train data. In the prediction of salinity and drought stress score, ReliefF, MRMR and F-test FS algorithms were used to select the appropriate subset of phenotype and genotype features. Each ML model was trained using features selected by FS algorithms. The performance of all ML models was evaluated by MAE, RMSE and R^2^ criteria. The model with the lowest MAE value over the test data set was selected as the best performed model. The results of salinity stress prediction using phenotype and genotype features are presented in Table 1. In this table, the performance of three FS algorithms and 5 ML models over the training and testing phases are presented. The obtained results showed that the trained models with phenotype and genotype features do not perform well in predicting the salinity and drought stress scores.

**Table 1:** Results of prediction of salinity and drought stresses using phenotype and genotype features.

Statistics	Salinity stress						Drought stress					
	Train			Test			Train			Test		
	MAE	RMSE	R^2^	MAE	RMSE	R^2^	MAE	RMSE	R^2^	MAE	RMSE	R^2^
Phenotype features												
Max	1.12	1.60	1.00	1.06	1.53	0.33	1.12	1.61	0.99	1.07	1.53	0.27
Min	0.01	0.00	0.02	0.94	1.18	0.00	0.32	0.13	0.02	0.96	1.22	0.00
Average	0.82	1.02	0.48	1.01	1.36	0.13	0.84	1.01	0.52	1.02	1.37	0.13
Genotype features												
Max	1.10	1.52	1.00	1.15	1.67	0.22	1.08	1.41	1.00	1.22	1.91	0.15
Min	0.01	0.00	0.00	1.09	1.50	0.00	0.04	0.00	0.00	1.09	1.61	0.00
Average	0.75	0.95	0.48	1.13	1.61	0.07	0.74	0.81	0.61	1.15	1.73	0.06

The performance of five ML models in predicting plant salinity stress using a combination of phenotype and genotype features is presented in Table 2. The results showed that the ReliefF algorithm and the NN model outperformed other models in the training and test phases. The MAE, RMSE and R^2^ values obtained for the NN model in the training phase were 0.0764, 0.0073, and 0.9999, respectively. In the test phase, this model had MAE, RMSE and R^2^ values equal to 0.1206, 0.0308 and 0.9995, respectively. As demonstrated in Table 2, ReliefF algorithm and the NN model performs best in predicting drought stress compared to other ML models. The results showed that the NN model had MAE, RMSE and R^2^ values of 0.04, 0.01 and 0.99 over the training phase and 0.07, 0.01 and 0.99 over the testing phase, respectively.

**Table 2:** Results of salinity and drought stress prediction using combination of phenotype and genotype features.

ML models	Salinity stress						Drought stress					
	Train			Test			Train			Test		
	MAE	RMSE	R^2^	MAE	RMSE	R^2^	MAE	RMSE	R^2^	MAE	RMSE	R^2^
ReliefF algorithm												
RF	0.15	0.03	0.99	0.45	0.31	0.96	0.04	0.03	1.00	0.38	0.26	0.97
SVM	0.99	1.45	0.20	0.99	1.40	0.07	0.96	1.46	0.21	0.96	1.25	0.26
NN	0.08	0.01	0.99	0.12	0.03	0.99	0.04	0.01	0.99	0.07	0.01	0.99
GP	0.13	0.03	0.99	0.50	0.46	0.90	0.06	0.01	0.99	0.49	0.49	0.88
DT	0.37	0.34	0.96	0.53	0.60	0.82	0.35	0.36	0.95	0.43	0.45	0.90
MRMR algorithm												
RF	0.11	0.02	0.99	0.35	0.20	0.98	0.10	0.01	1.00	0.38	0.23	0.97
SVM	0.93	1.33	0.33	0.91	1.28	0.22	0.91	1.20	0.45	0.89	1.13	0.38
NN	0.06	0.01	0.99	0.13	0.03	0.99	0.09	0.01	0.99	0.13	0.03	0.99
GP	0.09	0.01	0.99	0.53	0.62	0.82	0.02	0.01	0.99	0.43	0.53	0.86
DT	0.31	0.25	0.98	0.40	0.36	0.94	0.32	0.28	0.97	0.45	0.46	0.90
F-test algorithm												
RF	0.09	0.02	0.99	0.45	0.32	0.95	0.15	0.03	1.00	0.39	0.26	0.97
SVM	0.90	1.17	0.48	0.94	1.23	0.32	0.84	0.98	0.64	0.88	1.05	0.52
NN	0.05	0.01	0.99	0.14	0.05	0.99	0.09	0.01	0.99	0.13	0.03	0.99
GP	0.10	0.01	0.99	0.48	0.53	0.86	0.02	0.01	0.99	0.42	0.38	0.93
DT	0.30	0.26	0.97	0.45	0.45	0.90	0.23	0.19	0.99	0.41	0.43	0.91

**Figure 1:**
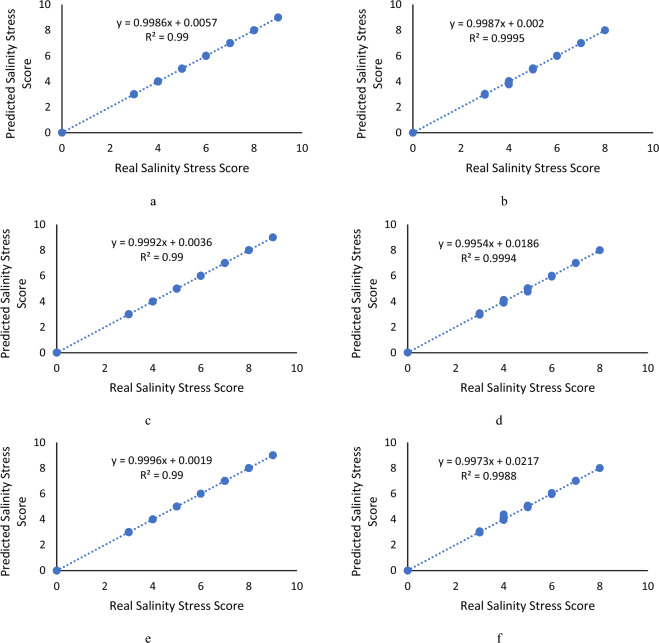
The regression results between the actual and predicted salinity stress values by NN model: (**a**) ReliefF algorithm over the train dataset, (**b**) ReliefF algorithm over the test dataset, (**c**) MRMR algorithm over the train dataset, (**d**) MRMR algorithm over the test dataset, (**e**) F-Test algorithm over the train dataset, (**f**) F-Test algorithm over the test dataset.

Comparing real and predicted salinity stress scores as well as drought stress, the regression equation and R^2^ of NN model over the train and test datasets are shown in Fig. 1. The training sample points are distributed near the perfect fit line (“actual stress scores = predicted stress scores”). The R^2^ values are above 0.98, indicating that the model can achieve high training effects. After the model training, the testing data set is used to verify and evaluate the model. As shown in this figure, by analyzing the correlation and error between the predicted stress scores and the actual stress scores of the test data set, it can be seen that the test sample points are also basically distributed in near the perfect fitted line (“actual stress scores = predicted stress scores”). The prediction performance of the model indicates that the prediction performance of the NN models is all reaching high prediction accuracy.

**Figure 2:**
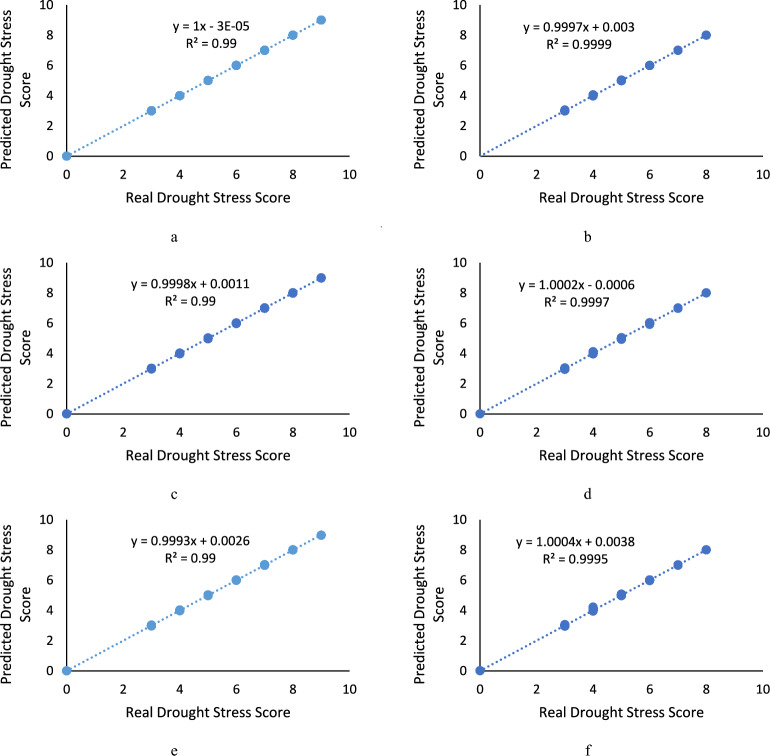
The regression results between the actual and predicted drought stress values by NN model: (**a**) ReliefF algorithm over the train dataset, (**b**) ReliefF algorithm over the test dataset, (**c**) MRMR algorithm over the train dataset, (**d**) MRMR algorithm over the test dataset, (**e**) F-Test algorithm over the train dataset, (**f**) F-Test algorithm over the test dataset.

Comparing real and predicted salinity stress scores as well as drought stress, the regression equation and R^2^ of NN model over the train and test datasets are shown in Fig. 2. The training sample points are distributed near the perfect fit line (“actual stress scores = predicted stress scores”). The R^2^ values are above 0.98, indicating that the model can achieve high training effects. After the model training, the testing data set is used to verify and evaluate the model. As shown in this figure, by analyzing the correlation and error between the predicted stress scores and the actual stress scores of the test data set, it can be seen that the test sample points are also basically distributed in near the perfect fitted line (“actual stress scores = predicted stress scores”). The prediction performance of the model indicates that the prediction performance of the NN models is all reaching high prediction accuracy.

### Classification of salinity and drought stress tolerance

The results of salinity stress tolerance classification by applying phenotype features are presented in Table 3. Using the accuracy index over the test dataset to compare the FS algorithms and ML models, the results showed that the ReliefF algorithm and the KNN model possessed accuracy, precision, and F1 score equal to 0.95, 0.96, and 0.95 in the training phase, and 0.91, 0.95, and 0.91 in the test phase respectively, outperformed other models. Table 3 also shows the results of salinity stress classification using genotype features. The results indicate that the ReliefF FS algorithm and the KNN model outperform other models with accuracy, precision and F1 score equal to 0.97, 0.98 and 0.97, in the training phase and 0.89, 0.89, and 0.89 in the testing phase respectively.

**Table 3:** Results of classification of salinity stress tolerance.

ML models	Phenotype features						Genotype features						Genotype and phenotype features					
	Train			Test			Train			Test			Train			Test		
	Accuracy	Precision	F1-score	Accuracy	Precision	F1-score	Accuracy	Precision	F1-score	Accuracy	Precision	F1-score	Accuracy	Precision	F1-score	Accuracy	Precision	F1-score
ReliefF algorithm																		
DT	0.84	0.83	0.85	0.71	0.68	0.73	0.71	0.72	0.70	0.63	0.63	0.63	0.91	0.94	0.91	0.76	0.75	0.77
DA	0.61	0.74	0.48	0.58	0.69	0.41	0.61	0.71	0.48	0.55	0.62	0.38	0.62	0.75	0.48	0.62	0.76	0.47
NB	0.83	0.87	0.82	0.71	0.69	0.72	0.76	0.78	0.75	0.66	0.66	0.66	0.87	0.91	0.87	0.78	0.79	0.78
SVM	0.95	0.95	0.95	0.73	0.71	0.74	0.78	0.82	0.77	0.61	0.62	0.60	0.90	0.88	0.90	0.79	0.75	0.80
KNN	0.95	0.96	0.95	0.91	0.95	0.91	0.97	0.98	0.97	0.89	0.89	0.89	0.98	0.97	0.98	0.96	0.98	0.96
RF	0.98	0.98	0.98	0.81	0.80	0.82	0.87	0.89	0.86	0.71	0.70	0.71	0.93	0.94	0.93	0.87	0.88	0.87
NN	0.87	0.89	0.87	0.72	0.72	0.72	0.68	0.71	0.66	0.60	0.61	0.58	0.85	0.87	0.84	0.76	0.77	0.76
MRMR algorithm																		
DT	0.91	0.90	0.91	0.66	0.65	0.68	0.72	0.70	0.73	0.60	0.59	0.62	0.87	0.87	0.87	0.72	0.71	0.73
DA	0.61	0.72	0.48	0.56	0.61	0.42	0.61	0.63	0.58	0.46	0.45	0.42	0.64	0.79	0.51	0.61	0.71	0.47
NB	0.81	0.85	0.80	0.64	0.59	0.72	0.82	0.85	0.81	0.63	0.58	0.71	0.84	0.88	0.83	0.71	0.73	0.70
SVM	0.96	0.96	0.96	0.81	0.79	0.82	0.99	0.99	0.99	0.72	0.68	0.74	0.99	0.99	0.99	0.85	0.82	0.85
KNN	0.97	0.98	0.97	0.84	0.82	0.85	0.90	0.88	0.90	0.76	0.74	0.77	0.99	0.98	0.99	0.98	0.99	0.98
RF	0.94	0.94	0.94	0.75	0.74	0.75	0.85	0.85	0.85	0.69	0.67	0.71	0.92	0.94	0.91	0.89	0.90	0.89
NN	0.93	0.93	0.93	0.74	0.72	0.76	0.71	0.73	0.70	0.65	0.65	0.66	0.97	0.98	0.97	0.77	0.76	0.78
Chi2 algorithm																		
DT	0.89	0.87	0.89	0.68	0.66	0.69	0.72	0.69	0.74	0.62	0.60	0.66	0.91	0.90	0.92	0.73	0.69	0.75
DA	0.62	0.75	0.48	0.59	0.71	0.42	0.61	0.61	0.61	0.46	0.47	0.48	0.64	0.79	0.51	0.61	0.71	0.47
NB	0.81	0.84	0.80	0.68	0.70	0.67	0.88	0.91	0.87	0.60	0.56	0.69	0.99	0.99	0.99	0.79	0.80	0.78
SVM	0.95	0.96	0.95	0.78	0.76	0.78	0.88	0.87	0.88	0.73	0.71	0.74	0.99	0.99	0.99	0.86	0.84	0.86
KNN	0.95	0.96	0.95	0.82	0.81	0.83	0.82	0.85	0.82	0.79	0.79	0.79	0.93	0.94	0.93	0.96	0.98	0.96
RF	0.99	0.99	0.99	0.81	0.84	0.80	0.82	0.82	0.82	0.72	0.72	0.73	0.91	0.90	0.92	0.86	0.87	0.86
NN	0.98	0.98	0.98	0.71	0.70	0.72	0.76	0.80	0.75	0.62	0.64	0.60	0.98	0.98	0.98	0.75	0.73	0.76

The results of salinity stress classification based on the combination of phenotype and genotype features are presented in Table 3. By comparison, the MRMR algorithm and KNN model performed better than other algorithms in the training and test phases. The results showed that the accuracy, precision, and F1 score of the KNN model were 0.99, 0.98, and 0.99, respectively. During the testing phase, the model has accuracy, precision, and F1 score values of 0.98, 0.99, and 0.98, respectively.

Obtaining similar results, the ReliefF algorithm and the KNN model classified drought stress tolerance with accuracy, precision, and F1 score equal to 0.99, 0.99, and 0.99 in the training phase, and 0.89, 0.89, and 0.90 in the test phase respectively (Table 4). The table shows that the KNN model performs better in classifying drought stress than other ML models. The comparison of FS algorithms also shows that the ReliefF algorithm has better results than other FS algorithms. The results show that the accuracy, precision and F1 score values of the KNN model are 0.99, 0.99 and 0.99 in the training phase and 0.85, 0.86 and 0.85 in the testing phase.

**Table 4:** Results of classification of drought stress tolerance.

ML models	Phenotype features						Genotype features						Genotype and phenotype features					
	Train			Test			Train			Test			Train			Test		
	Accuracy	Precision	F1-score	Accuracy	Precision	F1-score	Accuracy	Precision	F1-score	Accuracy	Precision	F1-score	Accuracy	Precision	F1-score	Accuracy	Precision	F1-score
ReliefF algorithm																		
DT	0.92	0.92	0.92	0.71	0.69	0.72	0.80	0.76	0.81	0.65	0.62	0.68	0.90	0.92	0.90	0.76	0.78	0.75
DA	0.61	0.74	0.48	0.59	0.71	0.42	0.61	0.71	0.48	0.55	0.62	0.38	0.61	0.73	0.47	0.61	0.73	0.48
NB	0.84	0.89	0.83	0.71	0.73	0.69	0.76	0.78	0.75	0.66	0.66	0.66	0.87	0.91	0.87	0.78	0.79	0.78
SVM	0.76	0.77	0.76	0.73	0.72	0.74	0.79	0.82	0.78	0.58	0.57	0.61	0.99	0.99	0.99	0.86	0.84	0.87
KNN	0.99	0.99	0.99	0.89	0.89	0.90	0.99	0.99	0.99	0.85	0.86	0.85	0.98	0.99	0.98	0.97	0.99	0.97
RF	0.91	0.94	0.91	0.72	0.72	0.73	0.83	0.84	0.82	0.66	0.66	0.67	0.99	0.98	0.99	0.86	0.86	0.86
NN	0.78	0.77	0.79	0.68	0.67	0.69	0.83	0.84	0.82	0.64	0.62	0.67	0.96	0.95	0.96	0.75	0.74	0.76
MRMR algorithm																		
DT	0.90	0.89	0.90	0.67	0.66	0.69	0.79	0.76	0.80	0.62	0.61	0.63	0.92	0.92	0.92	0.72	0.69	0.73
DA	0.62	0.76	0.49	0.57	0.65	0.42	0.60	0.73	0.45	0.52	0.56	0.33	0.64	0.79	0.51	0.61	0.71	0.47
NB	0.81	0.86	0.80	0.68	0.70	0.67	0.99	1.00	0.99	0.64	0.59	0.72	0.84	0.88	0.83	0.71	0.73	0.70
SVM	0.74	0.78	0.73	0.73	0.77	0.71	0.79	0.80	0.79	0.58	0.57	0.60	0.99	0.99	0.99	0.84	0.81	0.84
KN N	0.99	0.98	0.99	0.85	0.83	0.86	0.99	0.99	0.99	0.74	0.71	0.75	0.98	0.99	0.98	0.95	0.96	0.95
RF	0.94	0.95	0.94	0.69	0.68	0.70	0.80	0.77	0.80	0.61	0.59	0.63	0.97	0.98	0.97	0.82	0.82	0.83
NN	0.81	0.81	0.81	0.68	0.65	0.70	0.82	0.83	0.81	0.64	0.62	0.66	0.80	0.81	0.80	0.70	0.69	0.71
Chi2 algorithm																		
DT	0.92	0.92	0.92	0.72	0.70	0.73	0.79	0.84	0.78	0.66	0.70	0.62	0.92	0.89	0.92	0.74	0.70	0.77
DA	0.62	0.75	0.48	0.59	0.71	0.42	0.63	0.66	0.59	0.51	0.51	0.48	0.64	0.79	0.51	0.61	0.71	0.47
NB	0.99	0.99	0.99	0.74	0.73	0.75	0.81	0.84	0.80	0.68	0.70	0.67	0.99	0.99	0.99	0.79	0.80	0.78
SVM	0.80	0.84	0.79	0.65	0.65	0.64	0.78	0.83	0.76	0.55	0.56	0.48	0.99	0.99	0.99	0.84	0.81	0.84
KNN	0.98	0.98	0.98	0.86	0.86	0.87	0.98	0.97	0.98	0.84	0.84	0.84	0.98	0.98	0.98	0.96	0.98	0.96
RF	0.93	0.93	0.93	0.75	0.75	0.75	0.82	0.79	0.83	0.66	0.64	0.68	0.98	0.99	0.98	0.87	0.86	0.87
NN	0.74	0.76	0.73	0.65	0.67	0.63	0.80	0.82	0.79	0.61	0.62	0.58	0.94	0.96	0.94	0.78	0.82	0.77

Table 4 shows that the KNN model performs better than other ML models in classifying drought stress. A comparison of FS algorithms also shows that the ReliefF algorithm gives better results than other FS algorithms. The results showed that the values of accuracy, precision and F1 score of the KNN model are 0.99, 0.99 and 0.98 in the training phase and 0.97, 0.99 and 0.97 in the testing phase.

**Figure 3:**
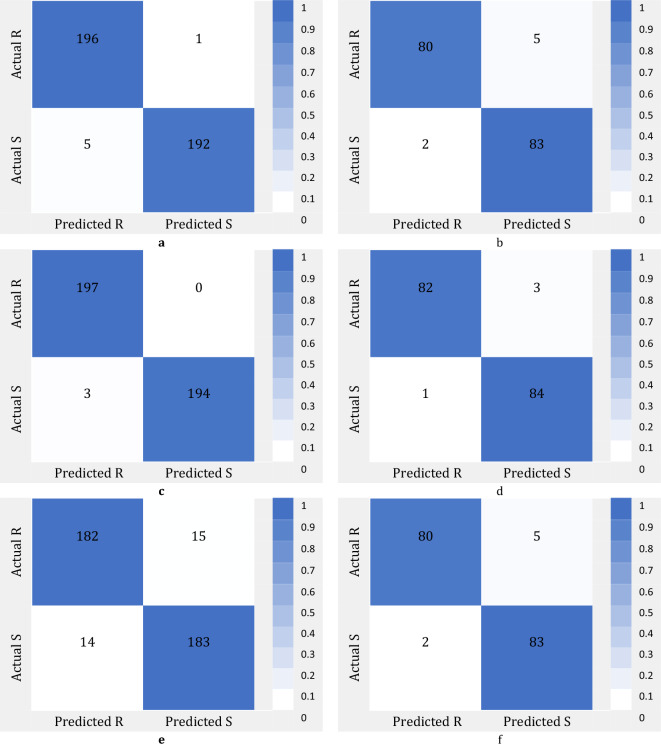
Confusion matrixes of KNN classifier for salinity stress using phenotype and genotype features: (**a**) ReliefF algorithm over train dataset; (**b**) ReliefF algorithm over test dataset; (**c**) MRMR algorithm over train dataset; (**d**) MRMR algorithm over test dataset; (**e**) Chi2 algorithm over train dataset; (**f**) Chi2 algorithm over test dataset.

We used the confusion matrix to demonstrate the performance details of ML models in classification of salinity and drought stress tolerance. A confusion matrix with multifaceted views is fundamental in evaluating classification performance. Confusion matrices were created for training and testing data sets. The data shown in the columns on the confusion matrix is related to the actual data and the data shown in the rows represents the classification results of the test data. The confusion matrixes of ML models in the classification of salinity stress during train and test stages are shown in Fig. 3. In this figure the confusion matrixes of KNN classifier and ReliefF, MRMR and Chi2 FS algorithms are presented.

**Figure 4:**
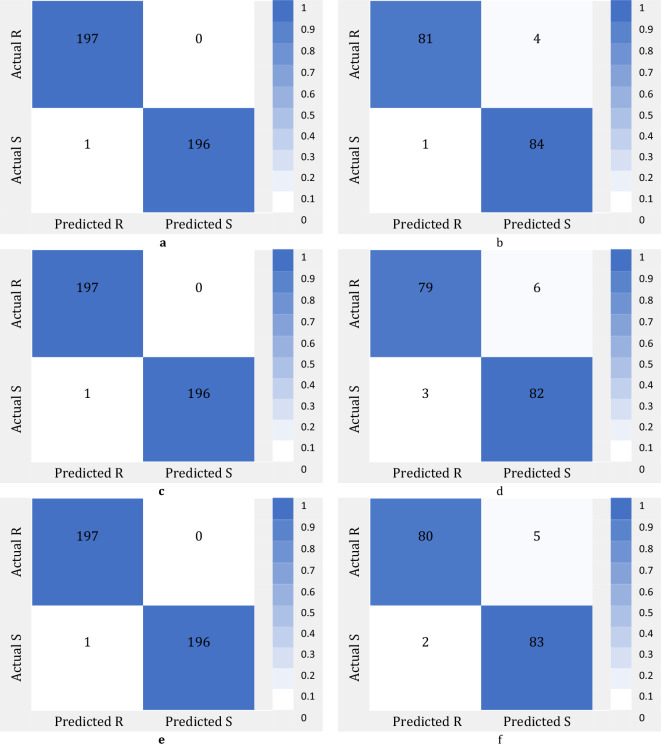
Confusion matrixes of KNN classifier for drought stress using Phenotype and Genotype Features: (**a**) ReliefF algorithm over train dataset; (**b**) ReliefF algorithm over test dataset; (**c**) MRMR algorithm over train dataset; (**d**) MRMR algorithm over test dataset; (**e**) Chi2 algorithm over train dataset; (**f**) Chi2 algorithm over test dataset.

The confusion matrixes of ML models in the classification of drought stress during train and test stages are shown in Fig. 4. In this figure the confusion matrixes of KNN classifier and ReliefF, MRMR and Chi2 FS algorithms are presented.

Selecting the KNN classifier as the best performing one in classification of salinity and drought stresses tolerance, the four basic ratio metrics including True Positive Rate (TPR), Positive Predicted Value (PPV), False Negative Rate (FNR) and False Discovery Rate (FDR) are shown in Table 5. Regarding TPR and PPV, during the test stage, the MRMR FS algorithm had the best performance in classification of salinity stress tolerance. Also, the ReliefF FS algorithm outperformed other algorithms in classification of drought stress tolerance. Considering FNR and FDR, the MRMR and ReliefF FS algorithms resulted in lowest classification error in classification of salinity and drought stresses tolerance respectively.

**Table 5:** Performance metrics for classification of Salinity and drought stress tolerance using KNN classifier.

FS algorithm	Train dataset				Test dataset			
	TPR	PPV	FNR	FDR	TPR	PPV	FNR	FDR
Salinity stress tolerance								
ReliefF	0.9949	0.9751	0.0051	0.0249	0.9412	0.9756	0.0588	0.0244
MRMR	1.0000	0.9850	0.0000	0.0150	0.9647	0.9880	0.0353	0.0120
Chi2	0.9239	0.9286	0.0761	0.0714	0.9412	0.9756	0.0588	0.0244
Drought stress tolerance								
ReliefF	1.0000	0.9949	0.0000	0.0051	0.9529	0.9878	0.0471	0.0122
MRMR	1.0000	0.9949	0.0000	0.0051	0.9294	0.9634	0.0706	0.0366
Chi2	1.0000	0.9949	0.0000	0.0051	0.9412	0.9756	0.0588	0.0244

## Discussion

During long-term exposure to drought stress, agricultural plants may be destroyed, or their production may be significantly reduced. Conversely, Soil salinity is an essential factor in reducing agricultural production. Carrying out agricultural operations to prevent salinization of fields, such as drainage, as well as planting perennial plants and low irrigation of fields, are a solution to deal with salinity. Drought and salinity stress tolerance were classified and predicted in this paper. The classification accuracy of salinity and drought stress was equal to 0.98 and 0.97, respectively. So, both stresses were classified with high accuracy. Achieving a high-accuracy model of stress classification will significantly help the lines resist drought and salt stress. In the prediction phase, the drought stress score was predicted better than the salinity score. However, the R^2^ of both stress predictions was 0.99. Since determining the stress score based on the plant's appearance requires an expert's knowledge and experience, stress score predictors reduce the dependence on individual senses and make the scoring process more precise.

This research shows the importance of using phenotype and genotype traits in stress tolerance modeling in barley lines. Using phenotype and genotype traits improved the ML model's performance compared to using phenotype traits alone. In the classification of salt stress resistance, accuracy, precision and f1 score were increased by 7.69%, 4.21% and 7.69%, respectively. This increase in drought stress classification equals 8.99%, 11.23% and 7.78%, respectively. The performance of the improved model in predicting the stress score has been much more impressive. In predicting salinity stress score, MAE and RMSE decreased by 88.11% and 97.74%, respectively, and R^2^ increased by 665.314%. In predicting the drought score, MAE and RMSE decreased by 92.83% and 99.23% respectively. Also, R^2^ increased by 667.972%.

Feature selection algorithms reduce the time and cost required for phenotype and genotype measurements. The ReliefF algorithm performed better in classification and prediction schemes than other FS algorithms. ReliefF is a filtering FS method inspired by instance-based learning. This algorithm is a well-known preprocessing method that can be used in many data mining problems. ReliefF effectively ranks features based on their quality. This algorithm can work on both nominal and numerical datasets. ReliefF estimates the degree of importance of features by calculating the difference between features. ReliefF can work on datasets with missing values and datasets with more than two categories of data. Instead of selecting one of the nearest neighbors done in the Relief algorithm, Relief finds a number of the nearest neighbors of a selected sample. ReliefF also uses a different function to calculate feature weights to handle incomplete data sets.

ML models' performance in the barley tolerance to drought and salinity classification showed that the KNN model gives much better results than other models. The KNN model is simple and cheap to implement, does not require a parameter estimation stage, is capable of nonlinear modeling, and is effective and works efficiently in dealing with many categories of data. This model can be one of the best options for multi-class classification due to its simplicity and lack of high complexity.

In the prediction section, the NN model outperformed other models. The NNs are examples of flexible regression approaches. However, they have fundamental differences from classical (parametric) techniques. No initial assumption regarding the model's shape is required in making the model. Solutions that provide for modeling complex nonlinear relationships are better than parametric models. They can deal with problems that include nonlinear relationships between variables. However, NNs cannot solve problems defined without uncertainty and are known as black box techniques. Uncertainty conditions often arise during the rapid development of new technologies, inaccurate and insufficient data, and the lack of confidence in the adequacy of defined independent variables. Two critical factors in adjusting and increasing or decreasing the error rate in the NN model are the number of hidden layers and units in each layer. The greater the number of hidden layers, the more flexible it is. Increased net shooting and accuracy Calculations increase; however, this number cannot be increased as much as desired because the problem may not converge to the correct answer.

## Conclusion

In this study, we proposed GenPhenML, a new approach to predict the resistance of barley cultivars to abiotic stress (drought and salinity), using ML models by combining molecular markers and phenotypic data. By finding the main molecular markers and selecting the best model, GenPhenML successfully predicted the stress score and the NN model showed MAE of 0.1206 and 0.0727, RMSE of 0 0.0308 and 0.0105 and R^2^ of 0.9995 and 0.99 for salinity and drought predictions, respectively. In addition, GenPhenML successfully classified barley cultivars into stress-tolerant and stress-sensitive categories with greater than 97% accuracy for both types of stress. These findings increase the potential of GenPhenML as a powerful tool for barley breeding programs to develop new varieties with stress tolerance and ultimately contribute to global food security.

## Materials and methods

### Data preparation

The phenotype and genotype properties of barley were determined utilizing its agronomic characteristics under saline and drought conditions. For stress score prediction, 1236 data samples were collected from barley lines and divided randomly to train and test datasets, each including 70% and 30% of the whole data. For stress tolerance classification, 1128 data samples were divided randomly to train (70%) and test (30%) datasets. The genotype and phenotype features of barley lines were determined utilizing their agronomic characteristics under saline and drought conditions. In the greenhouse at Gonbad Kavous University, 103 lines of F8 families resulting from Badia and Kavir crossings were examined using a completely randomized design with three replications. Planting was done in 5-kg soil capacity pots, with seven seedlings per line. The population was developed to present the plant genetic materials under the Gonbad Kavous University’s license. All the methods were performed in accordance with relevant guidelines and regulations. Table 6 shows some physical and chemical features of the soil.

**Table 6:** Soil physical and chemical properties of the experiment site (0–30 cm depth).

EC (ds/m)	pH	Neutral substances (%)	Organic carbon (%)	N (%)	Phosphorus (ppm)	Potassium (ppm)	Clay (%)	Silt (%)	Sand (%)
1.19	7.6	9.5	0.90	0.09	11.4	316	29	58	13

Drought stress was applied during the reproductive stage, with a moisture content of 0.8 field capacity equal to 20% by weight moisture. Every other week, irrigation was performed, and the moisture level was lowered to 9% by weight moisture. The soil moisture level was modified by assessing the amount of moisture lost and compensating with water (20%). Salinity stress was applied during the reproductive stage by irrigation with a salt chloride source of 16 dS.m-1. Weekly assessments of the salinity of the saturated extract in pots demonstrated a weekly increase of up to 10–17 (dS.m-1). The saturated extract was created by pouring 150 g of potting soil into a plastic bucket, adding distilled water, mixing, and shining the top. For phenotyping measurements, 15 competing plants of each line were measured, and their average was considered in the analysis. Phenotype scores were measured according to the protocols recommended by Chang and Yoshida^14,15^. The measurement instructions are provided in Tables 7 and 8.

**Table 7:** Instructions for drought tolerance.

Reaction	Leaf tubing	Leaf burn	Score
Highly tolerant	No signs of stress	No signs of stress	0
Tolerant	No leaf rolling	Partial drying of leaf tips	1
Moderately Tolerant	Partially rollingand no rolling in the morning	Dissipation of leaf tip dryness by a quarter in three leaves of the plant	3
Moderately Susceptible	Partially ruling and no ruling in the morning and evening	Drying of half of the young leaves and all the lower leaves	5
Susceptible	Fully rolling and no rolling in the morning	The dryness of the leaves spread to three-quarters of the leaves	7
Highly Susceptible	Like the roll and the rolling in the morning	Drought spread to all leaves	9

**Table 8:** Instructions for salinity stress tolerance.

Reaction	Damage	Score
Highly tolerant	Normal growth, no leaf symptoms	1
Tolerant	Nearly normal growth, but leaf tips or few leaves whitish and rolled	3
Moderately Tolerant	Growth severely retarded, most leaves rolled, only a few are elongating	5
Susceptible	Complete cessation of growth, most leaves dry, some plants dying	7
Highly Susceptible	Almost all plants dead or dying	9

The genotyping analysis was performed using crude DNA preparation. In a 1.5 ml centrifuge tube labeled with a label, a single leaf was extracted and placed in ice for a while. The leaf sample was macerated using 400 μl of extraction buffer (50 mM Tris–HCl, pH 8.0, 2.5 mM EDTA, 300 mM NaCl, and 1% SDS). It was ground until the buffer turned green. After that, 400 μl of extraction buffer was added and mixed by pipetting. For 10 min, the contents were centrifuged at 12,000 g in a microcentrifuge. Nearly 400 μl of lysate was extracted with 400 μl chloroform. The top supernatant was transferred to another 1.5ml tube, where DNA precipitation was performed with absolute ethanol. We centrifuged the contents for three minutes at full speed and discarded the supernatants. We rinsed the pellets with 70% ethanol and dried the DNA before resuspending it in 50 μl TE buffer (10 mM Tris–HCl, pH 8.0, 1 mM EDTA, pH 8.0). An aliquot of the solution was used for PCR analysis and the remaining solution was stored at –20°C.

For marker analysis, 365 SSR markers were properly spread over seven barley chromosomes^16^. Based on the polymorphic SSR primers, the DNA of each line was amplified using primers exhibiting polymorphism. The PCR was performed using a thermocycler (iCyclerBIORAD, USA) with template DNA 50 ng in 15 μl reaction mixture of primers 0.67 M, reaction buffer 10 μl, MgCl_2_ 2.5 mM, dNTPs 0.2 mM and Taq polymerase 0.5 U. PCR was performed at initial denaturation of 94°C for 5 min, 30 cycles of denaturation at 94°C for 1 min, annealing at 58°C for 1 min, elongation at 72°C for 1.5 min, and final extension at 72°C for 5 min then storage in a refrigerator at 4°C. Separation and visualization of the final product were performed with 6% polyacrylamide gel electrophoresis and stained silver. ISSR, iPBS, IRAP, SCoT and CAAT markers were employed for the parental investigation. When the band amplified in the first parent, scores of 1 and 3 were used for the presence and absence of the band, respectively. Scores of 2 and 4 were also utilized when the band was amplified in the second parent.

### Phenotype and genotype features

Phenotype data includes 15 phenotype features obtained from each plant by direct measurements. Genotype features consisted of 719 molecular markers determined by genetic measurements. These genotype features were used for the prediction of salinity and drought stress. Three FS algorithms (ReliefF, MRMR and F-test) were deployed to determine important genotype features.

### Feature selection

Over the past decades, data collection and storage advances have forced many sciences to face vast amounts of information. The FS algorithms reduce the dimensionality of the data by selecting appropriate subsets of the original features^17^ This paper used ReliefF, MRMR, F-test and Chi2 algorithms to select the appropriate number of features to train ML models.

Kira and Rendell formulated the original Relief algorithm inspired by learning by example^18^. As an evaluation filter algorithm, the ReliefF algorithm can detect feature dependencies. This algorithm uses the concept of nearest neighbors to obtain feature statistics. In addition, it retains the general advantages of filtering algorithms, such as high relative convergence speed and independence of the selected features from the induction algorithm. The *diff* function in the ReliefF algorithm calculates the difference in feature value *A* between two samples, *I*_*1*_ and *I*_*2*_, where *I*_*1*_ = *R*_*i*_ (*R*_*i*_ is the target) and *I*_*2*_ is *H* or *M*, in weighted updates. Bump identifies the two closest neighbor instances of the target. One with the same class called Close Hit (*H*) and one with the opposite class called Close Miss (*M*). For discrete features, the *diff* function is defined as follows^19^

1 $$diff\left( {A.I_{1} .I_{2} } \right) = \left\{ {\begin{array}{*{20}l} o \hfill & { if \;value\left( {A.I_{1} } \right) = value\left( {A.I_{2} } \right)} \hfill \\ 1 \hfill & {if\; otherwise } \hfill \\ \end{array} } \right.$$

Furthermore, for continuous features, *diff* is defined as:

2 $$diff\left( {A.I_{1} .I_{2} } \right) = \frac{{\left| {value\left( {A.I_{1} } \right) - value\left( {A.I_{2} } \right)} \right|}}{\max \left( A \right) - \min \left( A \right)}$$

The performance of the MRMR algorithm is based on the performance of mutual information between two feature spaces, which increases as the probability of sharing two feature vectors increases. Mutual information between two variables, *x* and *y*, is obtained according to Eq. 3 based on the probability density function^20^.

3 $$I\left( {x.y} \right) = \mathop \sum \limits_{y \in Y} \mathop \sum \limits_{x \in X} p\left( {x.y} \right)\log (\frac{{p\left( {x.y} \right)}}{p\left( x \right)p\left( y \right)})$$

In the maximum correlation method, FS requires (*I*) to have the highest value with class *c*. This trend shows the most significant dependence of feature *x* on class *c*. Maximum correlation is one of the optimal feature search methods, which is obtained by Eq. 4 based on the average value of all mutual information values between individual features *x*_*i*_ and class *c*.

4 $$\max D\left( {S.c} \right). D = \frac{1}{\left| S \right|}\mathop \sum \limits_{{x_{i} \in S}} I\left( {x_{i} ;c} \right)$$

According to Eq. 4, the characteristics most dependent on the class are selected; However, this dependency between functions can be considerable. Therefore, the mutual information between features is obtained per Eq. 5 to reduce duplications.

5 $$\min R\left( S \right). R = \frac{1}{{\left| S \right|^{2} }}\mathop \sum \limits_{{x_{j} ,x_{i} \in S}} I\left( {x_{i} ;x_{j} } \right)$$

To achieve the optimal property due to the minimum and maximum release ratio, the two equations, 4 and 5, are combined to obtain Eq. 6.

6 $$\mathop {\max }\limits_{{x_{j} \in X - Sm - 1}} \left[ {I\left( {x_{j} ;c} \right) - \frac{1}{m - 1} \mathop \sum \limits_{{x_{j} \in Sm - 1}} I\left( {x_{j} ;x_{i} } \right)} \right]$$

In this equation, *m* represents the number of elements selected from the feature set *S*, and *x* is the feature vector^20^.

The F-test is a statistical test that calculates the ratio of variances between the instances with the same target value called groups and within a group for a feature in one-way Analysis of Variance (ANOVA). It ranks features based on higher f-score values, indicating fewer distances within groups and more distances between groups. The f-score in this method is given by:^21^.

7 $$F-score = \frac{{{{var}}iance\; between \;groups}}{{{t{var}}iance\; whithin \;groups}}$$

where variance between groups is the variance between groups indicated by the target feature, and variance within a group is the sum of variances within each group.

The Chi2 FS algorithm was used for stress classification, with individual chi-square tests used to assess the independence of predictor variables from response variables. A small p-value indicates that a predictor variable depends on the response variable, making it an important feature^22^.

### ML models

This Section presents a brief description of all deployed ML models. The ML models are introduced more conceptually than mathematically. The mathematical explanations of models can be found in textbooks^23,24^.

#### Gaussian process regression (GPR)

The GPR regression model is a nonparametric statistical method for determining the relationship between independent and dependent variables. It uses latent variables, an explicit basis function, and unknown data parameters. The latent function reflects the statistical nature of the model and is determined by the kernel of the variance function. GPR models can provide accurate estimates with confidence intervals at any spatial point, capturing model predictions' uncertainties. The parser can also choose individual base features to preview and specify the model's appearance. Building and optimizing GPR models is a task that is doable with today's high-performance computing capabilities^25^.

### Linear discriminant analysis (LDA)

The discriminant analysis (DA) classification introduced by R. Fisher is one of the simplest and easiest classifiers. There are two types of DA classifiers: linear discriminant analysis (LDA) and quadratic discriminant analysis (QDA). In LDA classification, the decision surface is linear, while in QDA, the decision boundary is nonlinear^26^. Discriminatory characteristics create decision boundaries to distinguish between different classes in different areas. Thus, the input space is divided into regions, each bounded by some decision boundaries. A classifier is represented by decision function c or discrimination, where c is the number of classes. Decision functions are used to define decision boundaries between classes and regions or between regions of each class. Therefore, the discriminant function is used to determine the class label of the unknown pattern based on comparing several discriminant functions c and assigning the maximum score of the unknown pattern to the class label. Therefore, the discriminant function will have the highest value in the region compared to the other discriminant functions^27,28^.

#### Neural network (NN)

Neural networks (NN) are derived from biological neural systems. These models, with their natural and intelligent structure and appropriate modeling of the neurons in the human brain, try to simulate the behavior of brain neurons through defined mathematical functions and synaptic function in natural neurons through the calculated weights in the communication lines of neurons are artificially modeled. The structure of an NN consists of input, output and hidden layers, communication weights and activation transfer functions. The input layer is a transmission layer and a means to prepare and introduce data; the output layer includes the values predicted by the network and the hidden layers, which consist of processor nodes and the place of data processing^29^.

#### Naive Bayes (NB)

NB is a probabilistic classifier using Bayesian theory in complete independence. For classification problems, the NB model is powerful and intuitive. NB’s predictions are based on categories and Bayesian theory and assume that the predictors are conditionally independent. NB classifiers assume that the presence of one feature in a class is independent of the presence of another feature^30^.

#### Support vector machine (SVM)

SVM is a hybrid approach for reducing classification errors that combines estimation of convex hulls with differential error reduction. This loss reduction function evaluates unfavorable locations. SVM also uses the linear kernels as a tainted version of the Gaussian kernel to incorporate nonlinear maps of vector properties in ample space. SVM classification has a linear decision area, and while non-error core models have more flexible nonlinear decision-making contexts, linear SVM classifiers train errors faster than SVM models^31^.

#### Decision tree (DT)

The DTs are algorithms that generate decision rules based on the expected reduction in entropy when an element is sorted. They overstimulate data and have poor performance when applied to new datasets. For better results, they are frequently used in group contexts such as RFs^32^.

#### Random forest (RF)

A RF is a bag of DTs. Each DT is applied to a new training dataset obtained by random sampling, replacing the original dataset. In addition, some randomness is introduced into the decision tree construction: a subset of features is randomly selected for each decision branch of the DT. The RF prediction is given as the mean prediction of a single DT^33^.

#### K-nearest neighbor (KNN)

One of the classifiers used in this research is KNN. In this method, in the training stage, all samples in the input space are multidimensional vectors. This space is divided into category labels and the position of these points. Usually, the distance of the new sample to all the training samples is a suitable criterion to determine the category of the new and unknown sample. The distance of two samples is calculated as Euclidean, Manhattan, and Chebyshev. To determine the category of a new sample, the distance of this sample with all the samples stored in the memory is calculated, and the k samples with the smallest distance to the unknown sample are selected. The category label of most of these k samples is considered the category label for the unknown sample^34^ .

### Hyperparameter optimization

Bayesian Optimization Algorithm (BOA) is an effective method of general optimization of objective functions, the evaluation of which is costly^35^. BOA is proper when the user cannot access the functions' form and can only access noisy objective function estimates. In this paper, hyperparameter tuning of ML models is performed by BOA. The BOA was proposed by Pelikan et al., 1999^36^. This algorithm evolves a population of candidate solutions by building a Bayes network and then sampling it. In the BOA, the initial population is often randomly generated with a uniform distribution over all possible solutions. Each iteration of the BOA consists of four steps: First, using one of the selection methods, promising answers are selected from the current population. In the second step, a Bayes network is built to describe the population of promising answers. In the third step, new candidate answers are generated through sampling from the Bayes network. In the fourth step, the new candidate's answers are added to the previous answers and replace all or some of them. The steps are repeated until a termination condition is reached. The termination condition can be convergence to a single member, reaching a sufficiently good solution, or reaching a certain number of iterations. There are different ways to perform each step of the BOA. For example, the initial population can be generated randomly or by using initial knowledge related to the problem. The selection stage can be done using any standard selection method in evolutionary algorithms. Also, different algorithms can be used to build the Bayes network, and different criteria can be used to evaluate the quality of candidate models. The ML model parameters optimized by the BOA are presented in Table 9.

**Table 9:** HyperParameters of ML models optimized by bayesian optimization algorithm.

ML Model	Optimized Hyperparameters
RF	NumLearningCycles, LearnRate, MinLeafSize
SVM	BoxConstraint, KernelSclae, Epsilon
NN	number of layers, activation function, lambda and layer size
GPR	Sigma
DT	MinLeafSize, MaxNumSplit, VariablesToSample
KNN	NumNeighbors, Distance
NB	Width, Kernel
LDA	Delta, Gamma

### Evaluation metrics

The ML algorithms have two phases: training and testing. During the training phase, a model was created to predict the state of other samples, and their performance was measured by a set of tests in the second phase. In the testing phase, the goal is to evaluate the algorithm's performance from different aspects. The regression method has a set of data called training data that is pre-classified and has specific labels. The goal is to find a method, function or rule based on the characteristics of the training data to classify the data to be entered into the model in the future. The performance of all ML models was evaluated by MAE, RMSE and R^2^ metrics^37^.

8 $$MAPE = \frac{1}{n}\mathop \sum \limits_{i = 1}^{n} \left| {\frac{{y_{i} - \overline{{y_{i} }} }}{{y_{i} }}} \right|$$

9 $$MSE = \frac{1}{n}\mathop \sum \limits_{i = 1}^{n} \left( {y_{i} - \overline{{y_{i} }} } \right)^{2}$$

10 $$R^{2} = 1 - \frac{{\mathop \sum \nolimits_{i = 1}^{n} \left( {y_{i} - \overline{{y_{i} }} } \right)^{2} }}{{\mathop \sum \nolimits_{i = 1}^{n} \left( {y_{1} - y_{ave} } \right)^{2} }}$$

In these equations, ${y}_{i} and \overline{{y }_{i}}$ are predicted value and actual value, ${y}_{ave}$ is the average of data set values and *n* is the number of observations.

**Figure 5:**
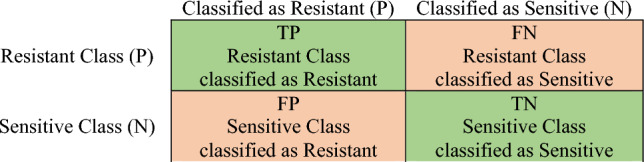
Confusion matrix, P: positive, N: negative, TP: true positive, FN: false negative, FP: false positive, TN: true negative.

In the case of classification, after training and testing the ML model, the confusion matrix on the training and testing dataset is computed to obtain the different types of misclassifications (Fig. 5). A confusion matrix contains information about different accuracy and error types. The confusion matrix is a matrix that shows the successful or unsuccessful performance of a classifier model. Each column of the matrix shows a sample of the value predicted by the model, and each row contains real (correct) samples. Confusion matrices make it easy to observe the error and interference between the results and are used to estimate the desired performance. The performance of a model is calculated by dividing the total number of elements of the main diagonal by the total number of elements of the matrix^38^.

The performance metrics for a multiclass confusion matrix are presented in Eqs. 11–15^39^.

11 $$Accuracy = \frac{TP + TN}{{TP + FP + TN + FN}}$$

12 $$Sensitivity = TP\; Rate \left( {TPR} \right) = \frac{TP}{{TP + FN}}$$

13 $$Specificity = TN\; Rate = \frac{TN}{{TN + FP}}$$

14 $$Precision = Positive\; Predictive\; Value \left( {PPV} \right) = \frac{TP}{{TP + FP}}$$

15 $$F1-Score = 2\frac{PPV \times TPR}{{PPV + TPR}} = \frac{{2n_{TP} }}{{2n_{TP} + n_{FN} + n_{FP} }}$$

## Data Availability

All data generated or analysed during this study are included in this published article.

## Abbreviations

FS: Feature selection
ML: Machine learning
QTLs: Quantitative trait loci
LDA: Linear discriminant analysis
NB: Naive Bayes
SVM: Support vector machine
KNN: K-nearest neighborhood
RF: Random forest
NN: Neural networks
GPR: Gaussian process regression
BOA: Bayesian optimization algorithm
MAE: Mean absolute error
RMSE: Root mean squared error
TP: True positive
FN: False negative
FP: False positive
TN: True negative
TPR: True positive rate
PPV: Positive predictive value
